# Fibrosis uncovered: ADAMTS12 cuts to the core of extracellular matrix drama

**DOI:** 10.1172/JCI183115

**Published:** 2024-09-17

**Authors:** Bernhard Dumoulin, Katalin Susztak

**Affiliations:** 1 Renal, Electrolyte, and Hypertension Division, Department of Medicine, University of Pennsylvania, Perelman School of Medicine, Philadelphia, Pennsylvania, USA.; 2 Institute for Diabetes, Obesity, and Metabolism, University of Pennsylvania, Perelman School of Medicine, Philadelphia, Pennsylvania, USA.; 3 Penn/CHOP Kidney Innovation Center, Philadelphia, Pennsylvania, USA.; 4 Department of Genetics, University of Pennsylvania, Perelman School of Medicine, Philadelphia, Pennsylvania, USA.

## Abstract

Fibrosis is a common manifestation of most progressive and degenerative diseases, with myofibroblast activation and matrix accumulation playing a key role. In this issue of the *JCI*, Hoeft et al. identify the important role of ADAMTS12 in fibroblast activation. ADAMTS12, a secreted protein, is involved in extracellular matrix (ECM) remodeling, cell signaling, and inflammation. ADAMTS12 facilitates proteolysis by cleaving various substrates such as ECM components, which are vital for cellular signaling and remodeling. Additionally, it modulates cell-matrix interactions, influencing cell adhesion and migration, and plays an important role in the inflammatory processes. Understanding the role of ADAMTS12 offers potential therapeutic insights for targeting fibrosis in progressive diseases.

## Fibrosis in heart and kidney disease

Fibrosis is a pathological process characterized by excessive accumulation of extracellular matrix (ECM) components, particularly collagen, in organs, including the heart and kidneys, often as a result of chronic injury or inflammation (1). While fibrosis has a protective role in the acute phase, targeting its chronic progression poses a therapeutic challenge and opportunity, with research focusing on developing treatments that prevent excessive ECM deposition without impairing initial healing responses (2). For example, after a myocardial infarction, the deposition of fibrotic matrix helps replace dead cells (e.g., cardiomyocytes in the heart) and preserve structural integrity, preventing rupturing and maintaining organ function (3). However, when the injury is repetitive or chronic, the continued accumulation of fibrotic tissue disrupts normal tissue architecture, leading to impaired organ function and eventually organ failure (3). In the heart, excessive fibrosis can stiffen the myocardium, impairing its ability to contract and pump blood effectively, leading to heart failure (3). In kidney disease, fibrosis manifests as glomerulosclerosis and tubulointerstitial fibrosis, where glomerulosclerosis involves ECM deposition within the glomeruli, obstructing the glomerular capillary tuft and impairing kidney function (4). Tubulointerstitial fibrosis on the other hand is characterized by the excessive deposition of ECM in the kidney interstitium, immune cell infiltration, and tubular atrophy (5). The decline in renal function seems to be strongly associated with tubulointerstitial fibrosis (6). Regardless of the underlying cause, fibrosis often marks the final stage of many chronic cardiovascular and renal diseases, making it a critical area of study for understanding and treating these conditions.

Recent human genetic studies have provided insights into the pathogenesis of kidney disease and fibrosis (7). The annotation of loci associated with low kidney function has highlighted genes that are predominantly expressed by kidney proximal tubule cells (7). A substantial portion of these newly identified genes are involved in modulating the metabolism of these tubule cells (8). The metabolic dysregulation observed in tubule cells not only causes a functional impairment but probably also contributes to cytokine and chemokine secretion by damaged epithelial cells (9). The severe mitochondrial damage observed in epithelial cells can lead to the cytosolic release of mitochondrial DNA and RNA, which in turn activate the cytosolic nucleotide–sensing mechanisms and the release of a range of cytokines, including TGF-β2, IL-34, CXCL10, CXCL1, and SPP1 among many (10, 11). Genetic variants can predispose proximal tubule cells to inflammatory cell death mechanisms, including pyroptosis and ferroptosis (10). These forms of cell death trigger an influx of immune cells, some of which may aid in the healing process, while others release cytokines that play crucial roles in tissue fibrosis (9). This process also includes activation of stromal cells and further injury of additional tubule and endothelial cells. This complex interaction between epithelial cells, immune cells, and fibroblast stromal cells creates a vicious cycle of progressive tissue damage and fibrosis, leading to a decline in kidney function over time. The interplay between these cell types exacerbates kidney damage and fibrosis, leading to a decline in kidney function over time.

## Characterization of stromal cells

While most cells are well characterized in the kidney, stromal cells (also called interstitial cells) represent a poorly characterized heterogenous population of cells. Recent single-cell gene expression analysis, however, has enabled the characterization of stromal cell types (12). It is generally agreed that stromal cells are positive for PDGFR-β expression. In the kidney, stromal cells include mesangial cells (which are positive for *ITGA8* and *POSTN*), vascular smooth muscle cells (VSMCs), pericytes (which are marked by *MYH11*, *NOTCH3*, and *NTRK3*), fibroblasts (which express *KCNK2* and *FAP*), and myofibroblasts (which express *COL1A1* and *SYNPO2*) (12). Subclustering analysis captured medullary fibroblasts expressing *SYT1* and *NCAM1* and four different myofibroblasts marked by *COL1A1*, *CLMP*, *FGF7*, or *ITGBL1* expression (12). Myofibroblasts are considered the main source of ECM during fibrogenesis (2). The ECM network plays a vital role as a stationary anchor for cellular adhesion within the fibrotic niche (13). ECM proteins are categorized as structural (including collagens, fibronectins, and elastin), matricellular (including fibrillin-1, tenascin-C, CTGF, and periostin), matrix-modifying proteins, and proteoglycans (13). Matricellular proteins are the most prevalent within the fibrotic kidney and can influence various cellular processes such as migration, apoptosis, ECM assembly, inflammation, wound healing, and fibrosis. These proteins serve as signal reservoirs and can aggregate growth factors and cytokines from the extracellular environment. Moreover, they influence cell behavior and serve as signal presenters by aiding the binding of ECM-associated ligands to their respective plasma membrane receptors.

Gli1^+^ perivascular cells were previously identified as important myofibroblast progenitors across major organs using genetic fate-tracing and ablation experiments (14). In this issue of the *JCI*, Hoeft, Koch, and co-authors isolated Gli1^+^ stromal cells from control mice and mice with unilateral ureter obstruction (UUO), a mouse model of kidney disease and fibrosis (15). Bulk RNA-Seq revealed that *Adamts12* was markedly upregulated in Gli1^+^ cells isolated from UUO mice (15). In humans, on the other hand, *ADAMTS12* expression was confined to a myofibroblast subset characterized by high *COL1A1* and *POSTN* expression (15), multiplex ISH demonstrated that *ADAMTS12* expression correlated with fibrosis and was specific to *PDGFRB*^+^ and *COL1A1*^+^ cells (15).

## A role for ADAMTS12 in fibrosis

A disintegrin and metalloprotease with thrombospondin motifs 12 (ADAMTS12) is a multifunctional protein that plays important roles in various physiological and pathological processes (16). It is involved in proteolysis, cell-matrix interactions, and inflammatory responses through its ability to cleave substrates such as ECM components and cytokines (16). ADAMTS12 is expressed in diverse tissues, with elevated levels observed during development and tissue remodeling (16). Notably, its expression is frequently upregulated in fibrotic conditions, such as liver and pulmonary fibrosis (16).

To investigate the role of ADAMTS12 in fibrosis in vivo, the authors compared UUO-induced kidney fibrosis in *Adamts12^–/–^* and control mice. *Adamts12^–/–^* mice showed decreased kidney fibrosis and lower expression of ECM proteins determined through mass spectrometry (15). In a myocardial infarction mouse model, *Adamts12^–/–^* mice exhibited reduced heart fibrosis and preserved left ventricular ejection fraction (LVEF) (15). Spatial transcriptomics compared gene expression in the ischemic zone, revealing decreased inflammatory signaling (i.e., *Nfkb*, *Tnfa*) and JAK/STAT signaling as well as enhanced cardiac muscle contraction and ECM remodeling pathways in *Adamts12^–/–^* mice (15). Predictive analysis of fibroblast cell states in the spatial data showed that knockout of *Adamts12* abrogated the expansion of epicardial and *Atf3^+^* injury-responsive fibroblasts (15).*ADAMTS12* expression was also observed in the ischemic zone of myocardial infarction in a human dataset (15). The authors then corroborated their findings with in vitro experiments in *ADAMTS12*-deficient PDGRFβ^+^ cells (15). They recapitulated the importance of ADAMTS12 on JAK/STAT signaling and additionally showed that *ADAMTS12* decreased migratory speed of fibroblasts in response to TGF-β (15). Furthermore, the decreased migratory speed could only be rescued by catalytically active ADAMTS12 (15). Interestingly, *ADAMTS12*-knockout cells exhibited altered ECM composition after TGF-β stimulation, with upregulation of fibulin and hemicentin 1 (HMCN1) (15). They subsequently showed that ADAMTS12 cleaved HMCN1. Knockdown of *HMCN1* in *ADAMTS12*-knockout cells inhibited migration, leading the authors to suggest that HMCN1 anchors pericytes and must be cleaved by ADAMTS12 to enable migration and fibroblast differentiation (15).

## Conclusions and next steps

The study by Hoeft et al. raises several important questions and potential next steps for further research. One key question is the precise mechanism by which ADAMTS12 modulates JAK/STAT signaling and how this interaction influences fibrosis and inflammatory responses. Future investigations will be needed to unravel the interplay between the injured tubular compartment, the perivascular niche, and ECM-producing myofibroblasts. In this regard, higher-resolution spatial transcriptomics (i.e., single-cell level) seems ideally suited. Additionally, investigating the role of ADAMTS12 in other fibrotic and inflammatory diseases beyond kidney and heart fibrosis could broaden its clinical relevance. Another important area for future research is the identification of specific inhibitors of ADAMTS12 and the testing of their efficacy and safety in preclinical and clinical settings. Furthermore, exploring the interaction between ADAMTS12 and other ECM components, such as fibulin and HMCN1, in different cellular contexts might reveal new targets for therapeutic intervention. Finally, longitudinal studies to track the progression of fibrosis and the effect of ADAMTS12 inhibition over time will be crucial in understanding the long-term benefits and potential side effects of such treatments.

**Figure 1:**
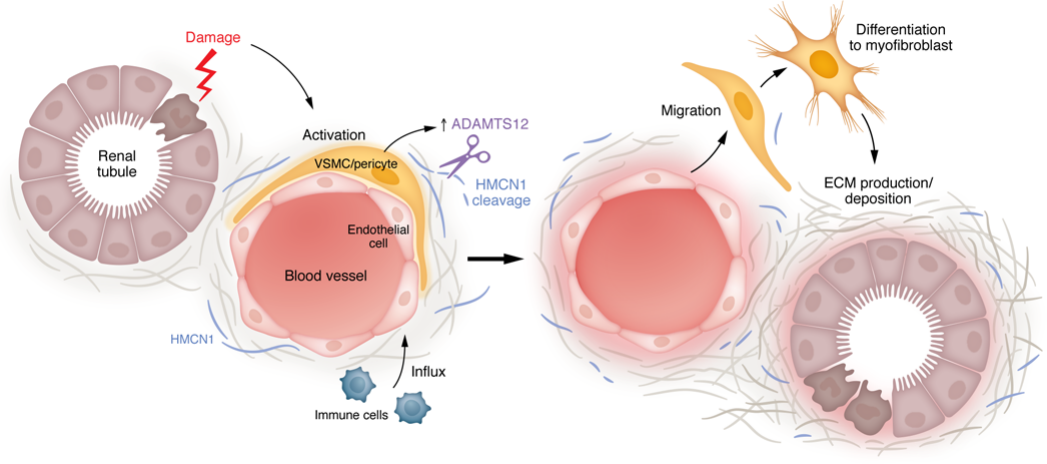
A proposed pathomechanism. Kidney damage to the tubular compartment initiates a cascade of pathological events, beginning with epithelial injury and the expression of cytokines, which result in an influx of immune cells and the activation of pericytes. Once activated, pericytes upregulate ADAMTS12, a metalloproteinase that plays a critical role in the degradation of ECM components. Specifically, ADAMTS12 mediates the cleavage of the large ECM protein HMCN1. Subsequently, pericytes migrate from the perivascular space and differentiate into myofibroblasts, key effector cells in fibrosis that are responsible for the excessive production and deposition of ECM. Additionally, the activation of pericytes and subsequent myofibroblast differentiation can perpetuate a cycle of chronic inflammation and tissue remodeling, exacerbating the progression of disease.

In summary, this study elegantly uncovers a mechanism by which pericytes, through upregulation of ADAMTS12, are enabled to detach, migrate from the perivascular niche, and differentiate into ECM-producing myofibroblasts (Figure 1). Especially in the context of myocardial fibrosis, in which ADAMTS12 loss not only reduced fibrosis but also preserved LVEF, ADAMTS12 inhibition could be an interesting therapeutic approach.

## Version 1 09/17/2024

Electronic publication
